# Risk Factors and Clinical Features of Deterioration in 739 COVID‐19 Affected Children Aged Under 14 Years in Zhuhai, China: A Multicenter, Retrospective Study

**DOI:** 10.1002/pdi3.70001

**Published:** 2025-05-19

**Authors:** Ting Liu, Zhaojun Pan, Cheng Zhang, Fanlin Huang, Jundong Ding, Wenxiu Song, Yongwu Xie, Guang Lin

**Affiliations:** ^1^ Department of Pediatric Rheumatology and Immunology Zhuhai Center for Maternal and Child Health Care Zhuhai Guangdong China; ^2^ Department of Pediatrics Zhuhai People's Hospital Zhuhai Guangdong China; ^3^ Department of Pediatrics Xiangzhou District People's Hospital Zhuhai Guangdong China; ^4^ Department of Pediatrics Zhuhai Fifth People's Hospital Zhuhai Guangdong China; ^5^ Department of Pediatrics The Fifth Affiliated Hospital of Zunyi Medical University (Zhuhai) Zhuhai Guangdong China

**Keywords:** children, clinical features, risk factor, SARS‐CoV‐2 infection

## Abstract

To retrospectively analyze the clinical characteristics of children with severe acute respiratory syndrome coronavirus 2 (SARS‐CoV‐2) infection, this study involved 739 children with SARS‐CoV‐2 infection who were admitted to multiple medical institutions in Zhuhai City from December 15, 2022 to January 24, 2023. Epidemiological and clinical data were collected and analyzed using statistical methods to understand the disease characteristics. The onset and progression of SARS‐CoV‐2 infection in children were significantly associated with age distribution, basic illness, vaccination status, exposure history, and family clustering. The most common clinical symptoms were fever (91.7%) and cough (81.6%). Laboratory findings indicated elevated neutrophil count, neutrophil‐to‐lymphocyte ratio, platelet‐to‐lymphocyte ratio, prothrombin time, and procalcitonin, creatine kinase, D‐dimer, and IL‐6 levels, along with decreased lymphocyte count, platelet count, and lymphocyte‐to‐monocyte ratio. Lung computed tomography (CT) showed imaging changes strongly linked to severe infection. After receiving anti‐inflammatory, symptomatic, supportive, and antipathogen therapies, 74% of the children displayed clinical symptomatic relief, 26% of the children were cured and discharged, and there were no fatalities. In Zhuhai, children infected with SARS‐CoV‐2 commonly exhibit family‐based transmission, with fever and cough as the predominant symptoms. Factors, such as young age, basic illness, specific laboratory markers, and patchy exudative changes on lung CT scans, served as critical indicators of severe infection. Early detection and monitoring of these factors, along with timely vaccination against novel coronavirus, can mitigate disease severity and prevent progression.

## Graphical abstract

Seven hundred and thirty‐nine children infected with SARS‐CoV‐2 in Zhuhai tend to have close‐contact aggregated morbidity within the family, with fever and cough as the main manifestations. Low age, underlying disease, some laboratory indicators, and patchy exudative changes on CT of the lungs are the windsocks of the severity of the infection.

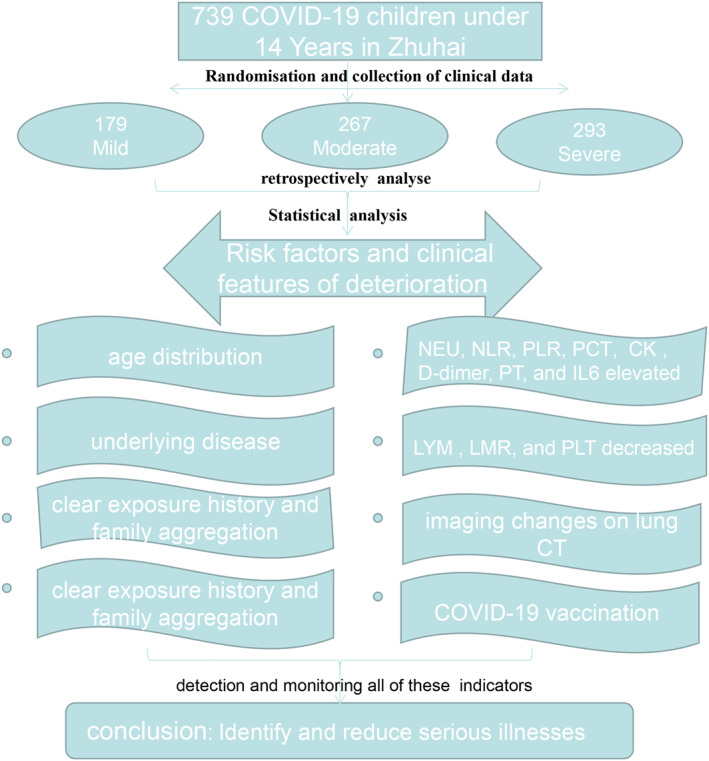

## 1 Introduction

Since December 2019, the outbreak of coronavirus disease 2019 (COVID‐19) originating in Wuhan has profoundly impacted the world [1]. Over the past 3 years, the virus has continually evolved and mutated, with new variants emerging frequently. Recently, the Omicron variant has become the dominant strain globally. Omicron is characterized by a shorter incubation period, higher infectivity, and faster replication in the bronchi when compared to the original strain, despite its diminished virulence and significant immune escape properties [2].

The incidence of infections among children, who are still developing and lack fully mature immune systems, has dramatically increased. The potential long‐term harm to affected children necessitates urgent attention from the pediatric community. On December 7, 2022, it was announced that the country's COVID‐19 restrictions would be lifted starting the next day, marking a new phase in outbreak prevention and control. This action led to the rapid spread of the Omicron variant, resulting in peaks of infections across several cities, including Zhuhai. On December 26, 2022, the National Health Commission renamed “novel coronavirus pneumonia” to “novel coronavirus infection,” reflecting the disease caused by the severe acute respiratory syndrome coronavirus 2 (SARS‐CoV‐2) Omicron variant.

This study retrospectively analyzed the epidemiological and clinical data of 739 cases admitted to multiple medical institutions in Zhuhai City during the large‐scale epidemic from December 15, 2022 to January 24, 2023. The objective was to summarize the clinical features, laboratory index characteristics, and treatment outcomes of these cases, so as to provide valuable insights into the prevention, control, and treatment of SARS‐CoV‐2 infections in children.

## 2 Materials and Methods

### 2.1 Study Subjects

Children aged 0–14 years with SARS‐CoV‐2 infection, who were admitted to multiple medical institutions in Zhuhai from December 15, 2022 to January 24, 2023, were included in this study. The inclusion criteria comprised COVID‐19 cases confirmed in designated hospitals, following the diagnostic criteria outlined in the Novel Coronavirus Pneumonia Diagnosis and Treatment Program (Trial 10th Edition) issued by the National Health Commission [3, 4], with a positive nucleic acid test for SARS‐CoV‐2. Clinical data were collected from all children with confirmed cases. Clinical classifications included mild, moderate, severe, and critical types:

Mild type: Mild clinical symptoms and no pneumonia detected on imaging.

Moderate type: Persistent high fever for more than 3 days, cough and shortness of breath, with a respiratory rate (RR) of < 30 breaths/min and oxygen saturation > 93% in room air. Imaging revealed characteristic pneumonia manifestations of new coronavirus infection.

Severe type (children presenting with any of the following symptoms): Ultrahigh fever or persistent high fever for more than 3 days; shortness of breath (RR ≥ 60 breaths/min for < 2 months of age, RR ≥ 50 breaths/min for 2–12 months of age, RR ≥ 40 breaths/min for 1–5 years of age, and RR ≥ 30 breaths/min for > 5 years of age), excluding effects of fever and crying; oxygen saturation ≤ 93% on room air at rest; nasal flaring, triple concave sign, wheezing, or stridor; impaired consciousness or convulsions; and refusal of food or difficulty feeding with signs of dehydration.

Critical type (children presenting with any of the following symptoms): Respiratory failure requiring mechanical ventilation; shock; and multiorgan failure requiring ICU supervision and treatment.

The mild type was classified as the mild group, the moderate type was classified as the moderate group, and the severe and critical types were classified as the severe group.

The exclusion criteria included children infected outside Zhuhai, those whose nucleic acid test did not meet the criteria for release from isolation by the time of discharge, and those with incomplete clinical data. The study received an ethical review waiver from the Ethics Committee of Zhuhai Center for Maternal and Child Health Care, number 2024 (102). Samples were collected after prior informed consent of the children or their parents/guardians.

### 2.2 Methods

A case information questionnaire was created using Excel. The electronic medical record system was used to collect data on sex, age, epidemiological history, underlying diseases, vaccination history, clinical manifestations, laboratory examinations, imaging, treatment plans, and prognosis data for 739 infected cases admitted to multiple healthcare institutions in Zhuhai City from December 15, 2022, to January 24, 2023. The research center distributed a uniform data collection form to five sentinel hospitals (Zhuhai Center for Maternal and Child Health Care, Zhuhai People's Hospital, Xiangzhou District People's Hospital, The Fifth Affiliated Hospital of Zunyi Medical University, and Zhuhai Fifth People's Hospital) that admitted infected cases. The head of each subresearch center included cases and extracted information from the medical record system. After completing data collection and verification, the subresearch centers sent the data to the research center for summarizing.

The first laboratory test results collected from all diagnosed patients included the following aspects: 1. Routine blood tests: white blood cell (WBC) count, neutrophil (NEU) count, lymphocyte (LYM) count, monocyte count (MO), platelet (PLT) count, and hemoglobin (Hb). 2. Inflammatory markers: erythrocyte sedimentation rate (ESR), C‐reactive protein (CRP), and procalcitonin (PCT). 3. Liver function tests: aspartate aminotransferase (AST), alanine aminotransferase (ALT), and albumin (ALB). 4. Cardiac function tests: Creatine kinase (CK), creatine kinase isoenzyme (CK‐MB), lactate dehydrogenase (LDH), and brain natriuretic peptide (BNP). 5. Renal function tests: Creatinine and urea nitrogen. 6. Coagulation tests: Fibrinogen (FIB), activated partial thromboplastin time (APTT), prothrombin time (PT), and D‐dimer (DD). 7. Cytokines: Interleukin‐6 (IL‐6). 8. Other tests: Blood glucose and immunoglobulin G (IgG) levels.

Additionally, the results of the first chest CT examination after admission were collected.

### 2.3 Statistical Analysis

Data from the mild, moderate, and severe groups were collated and analyzed using SPSS 22.0 (SPSS Inc., Chicago, IL, USA), and descriptive statistics, such as mean and standard error, were employed for within‐group comparisons and between‐group comparisons that were conformed to continuous variables with normal distribution. Depending on the assumption of normality and homogeneity of variance, a one‐way analysis of variance or the Kruskal–Wallis test was applied. Counting data were expressed as the number of cases and percentage, whereas the statistical association between categorical variables was obtained using chi‐squared tests or Fisher's exact tests. Spearman's correlation coefficient was applied to assess the correlation among variables, such as WBC, NEU, neutrophil‐to‐lymphocyte ratio (NLR), PLT, platelet‐to‐lymphocyte ratio (PLR), IL‐6, the liver, heart, kidney, and coagulation function, and inflammatory indexes (PCT, ESR, and CRP). *p* < 0.05 was considered to indicate statistical significance, and all *p* values were corrected to exclude other confounders.

## 3 Results

### 3.1 Analysis of Demographic Characteristics, Clinical Features, and Laboratory Results of Children Affected With SARS‐CoV‐2

#### 3.1.1 Demographic Characteristics

Table 1 shows the age distribution, sex, epidemiology, vaccination status, and underlying diseases of all participants. This study included 739 children with SARS‐CoV‐2 who were selected in accordance with strict inclusion and exclusion criteria. Children with mild, moderate, and severe diseases accounted for 24.2% (179), 36.1% (267), and 39.7% (293) of all cases, respectively. Among the children, 43.2% were of age < 1 year at the time of diagnosis, 38.3% were aged 1–3 years, 10.1% were aged 4–6 years, and 8.4% were aged 7–14 years. There was a statistically significant difference in the age distribution factor between these groups (*p* < 0.001). The proportion of patients within the 3‐year age group was 81.5%. Regarding sex distribution, males were generally more in numbers than females in each disease severity group, with an overall ratio of 1.64:1, although this difference was not statistically significant (*p* = 0.267). A significant proportion of cases had a known history of exposure (63.1%, *p* < 0.001) and family cluster morbidity (family cluster morbidity, defined as the occurrence of two or more cases within 2 weeks due to close contact or reciprocal infections within families, were strongly linked to the onset and progression of SARS‐CoV‐2 infection in children, 46.0%, and *p* = 0.004), indicating statistically significant differences between different severity groups. Only 5.6% of children had been vaccinated, with no statistically significant difference in the vaccination history observed among the severity groups (*p* = 0.22). The prevalence of basic disease (i.e., basic metabolic disorders, immunocompromised diseases, and major chronic wasting diseases) was 8.3%, with a statistically significant difference between the severity groups (*p* = 0.044).

**TABLE 1:** Demographic and epidemiological characteristics of 739 SARS‐CoV‐2 cases in Zhuhai.

Characteristics	Total case	SARS‐CoV‐2 *case*			*χ* ^*2*^ */p*
		Mild	Moderate	Severe	
Cases (*n*/[%])	739 (100%)	179 (24.2)	267 (36.1)	293 (39.7)	
Sex (male/female)	459/280	102/77	170/97	187/106	0.267
Age, years (*n*/[%])					
< 1	319 (43.2)	85 (11.5)	136 (18.4)	98 (13.3)	< 0.001
1–3	283 (38.3)	54 (7.3)	77 (10.4)	152 (20.6)	
4–6	75 (10.1)	17 (2.3)	35 (4.7)	23 (3.1)	
7–14	62 (8.4)	23 (3.1)	19 (2.6)	20 (2.7)	
Exposure history (*n*/[%])					
Yes	466 (63.1)	117 (15.8)	188 (25.4)	161 (21.9)	< 0.001
No	159 (21.5)	36 (4.9)	39 (5.3)	84 (11.4)	
Unknown	114 (15.4)	26 (3.5)	40 (5.4)	48 (6.5)	
Family cluster morbidity (*n*/[%])					
Yes	340 (46.0)	103 (13.9)	122 (16.5)	115 (15.6)	0.004
No	245 (33.1)	48 (6.5)	86 (11.6)	111 (15.0)	
Unknown	154 (20.8)	28 (3.8)	59 (8.0)	67 (9.0)	
Vaccination (*n*/[%])	41 (5.6)	16 (2.2)	13 (1.8)	12 (1.6)	0.22
First	41 (5.5)	16 (2.2)	13 (1.8)	12 (1.6)	
Second	40 (5.4)	16 (2.2)	12 (1.6)	12 (1.6)	
Third	0	0	0	0	
No	558 (75.5)	129 (17.5)	206 (27.9)	223 (30.1)	
Unknown	140 (18.9)	34 (4.6)	48 (6.5)	58 (7.8)	
Basic disease (*n*/[%])					
Yes	61 (8.3)	21 (2.8)	14 (2.0)	26 (3.5)	0.044
No	678 (91.7)	158 (21.4)	253 (34.2)	267 (36.1)	

*Note:* The counting data were expressed as the number of cases and percentage, and the comparisons among the mild, moderate, and severe groups were made using the *χ2* test. Basic diseases mainly include basic metabolic disorders, immunocompromised diseases, and major chronic wasting diseases.

The significance value was set at *p* < 0.05.

#### 3.1.2 Clinical Manifestations

The most common clinical manifestations observed in children with SARS‐CoV‐2 infection were fever (91.6%), cough (81.6%), runny nose (53.7%), nasal congestion (49.8%), convulsions (25.8%), sore throat (17.3%), hoarseness (18.7%), shortness of breath (10.6%), vomiting (10.6%), diarrhea (5.1%), abdominal pain (3.2%), hypoxemia (3.8%), dizziness and headache (1.9%), chest pain (1%), myalgia (1.2%), conjunctivitis (0.6%), hypogeusia (0.1%), and hyposmia (0.1%). The duration of fever, severity of cough, nasal congestion, runny nose, sore throat, hoarseness, and pulmonary rales varied significantly between the severity groups as detailed in Table 2 or Table S1. Notably, shortness of breath, convulsions, hypoxemia, hypogeusia, and respiratory failure were prominent manifestations among the severe group.

**TABLE 2:** Clinical characteristics of 739 SARS‐CoV‐2 cases in Zhuhai.

Characteristics	Total case (*n*/[%])	SARS‐CoV‐2 case			*χ2/p*
		Mild (*n*/[%])	Moderate (*n*/[%])	Severe (*n*/[%])	
Type case (*n*/[%])	739 (100%)	179 (24.2)	267 (36.1)	293 (39.7)	—
Fever case (*n*/[%])	677 (91.6)	152 (20.6)	250 (33.8)	275 (37.2)	—
Duration of fever (d, $\bar{x}$ ± s)	2.77 ± 1.97	2.4 ± 1.60	3.41 ± 2.32	2.39 ± 1.63	< 0.001
Severity of cough (*n*/[%])	603 (81.6)	131 (17.7)	254 (34.4)	218 (29.5)	
Single cough	268 (36.3)	70 (9.5)	73 (9.9)	125 (16.9)	< 0.001
Continuous cough	335 (45.3)	61 (8.3)	181 (24.5)	93 (12.6)	
Nasal congestion (*n*/[%])	368 (49.8)	82 (11.1)	163 (22.1)	123 (16.6)	< 0.001
Runny nose (*n*/[%])	397 (53.7)	89 (12.0)	165 (22.3)	143 (19.4)	0.004
Sore throat (*n*/[%])	128 (17.3)	44 (6.0)	13 (1.8)	71 (9.6)	< 0.001
Convulsion (*n*/[%])	191 (25.8)	0	0	191 (25.8)	—
Single/multiple	140 (18.9)/51 (6.9)	0	0	140 (18.9)/51 (6.9)	
5 min	148 (20.0)	0	0	148 (20.0)	
5min–30 min	29 (3.9)	0	0	29 (3.9)	
> 30 min	7 (0.01)	0	0	7 (0.01)	
Hoarseness (*n*/[%])	138 (18.7)	29 (3.9)	39 (5.3)	70 (9.5)	0.012
Shortness of breath (*n*/[%])	78 (10.6)	0	0	78 (10.6)	—
Abdominal pain (*n*/[%])	24 (3.2)	8 (1.1)	9 (1.2)	7 (1)	0.46
Diarrhea (*n*/[%])	38 (5.1)	14 (1.9)	12 (1.6)	12 (1.6)	0.172
Vomiting (*n*/[%])	78 (10.6)	25 (3.4)	27 (3.7)	26 (3.5)	0.21
Chest pain (*n*/[%])	8 (1.0)	4 (0.5)	3 (0.4)	1 (0.1)	0.153 (*Fisher's*)
Myalgia (*n*/[%])	9 (1.2)	3 (0.4)	3 (0.4)	3 (0.4)	0.82 (*Fisher's*)
Headache/dizziness (*n*/[%])	14 (1.9)	6 (0.8)	2 (0.3)	6 (0.8)	0.126 (*Fisher's*)
Hypogeusia (*n*/[%])	1 (0.1)	0	0	1 (0.1)	—
Hyposmia (*n*/[%])	1 (0.1)	0	0	1 (0.1)	—
Conjunctivitisv (*n*/[%])	5 (0.6)	3 (0.4)	1 (0.1)	1 (0.1)	0.23 (*Fisher's*)
Pulmonary rales (*n*/[%])					
Normal	424 (57.4)	136 (18.4)	84 (11.4)	204 (27.6)	< 0.001
Rale sputum	98 (13.3)	14 (1.9)	51 (6.9)	33 (4.5)	
Moist rale	214 (28.9)	24 (3.2)	135 (18.3)	55 (7.4)	
Hypoxemia (*n*/[%])	28 (3.8)	0	0	28 (3.8)	—
Respiratory failure (*n*/[%])	8 (1)	0	0	8 (1)	—

*Note:* The counting data were expressed as the numbers of cases and percentage, and the comparisons among the mild, moderate, and severe groups were made by the *χ2* test or Fisher's exact probability method.

The significance value was set at *p* < 0.05.

#### 3.1.3 Analysis of Laboratory Examination Results of Children With SARS‐CoV‐2

Table 3 and Table S1 show the results of laboratory tests, including routine blood tests, liver function tests, renal function tests, cardiac function markers, and inflammatory factors, including coagulation function, IL‐6, and D‐dimer, as well as IgG levels. In terms of laboratory findings, there was no statistically significant difference in the absolute WBC values among the patient groups. However, several parameters revealed significant differences. The absolute values of NEU count, NLR, PLR, PCT, and CK levels were significantly elevated in the severe group compared to those in both the mild (*p* < 0.001, *p* = 0.001, *p* = 0.016, *p* < 0.001, and *p* = 0.005, respectively) and moderate groups (*p* < 0.001, *p* < 0.001, *p* < 0.001, *p* < 0.001, and *p* < 0.001, respectively). Compared with the mild group and the moderate group (*p* = 0.941 and *p* = 0.941, respectively), the level of IL‐6 in the severe group also showed an increasing trend. Conversely, the absolute values of LYM, LYM‐to‐monocyte ratio (LMR), ESR, CK‐MB, FIB, and PLT were lower in the severe group than those in both the mild (*p* = 0.097, *p* = 0.142, *p* = 0.179, *p* = 0.217, *p* = 0.790, and *p* = 0.048, respectively) and moderate groups (*p* < 0.001, *p* < 0.001, *p* < 0.001, *p* < 0.001, *p* = 0.007, and *p* < 0.001, respectively). DD and PT were higher in the severe group than those in both the mild (*p* = 0.399 and *p* = 0.232, respectively) and moderate groups (*p* = 0.001 and *p* = 0.011, respectively). LYM, LMR, CRP, ESR, and FIB levels were reduced in the mild group compared to those in the moderate group (*p* = 0.001, *p* = 0.005, *p* < 0.001, *p* = 0.010, and *p* = 0.015, respectively). In the moderate group, LDH levels were significantly higher than in the mild and severe groups (*p* = 0.024 and *p* = 0.001, respectively). No statistically significant differences were observed among the groups for the remaining parameters.

**TABLE 3:** Laboratory results of 739 cases of SARS‐CoV‐2 in Zhuhai.

Clinical indicators	Total case ($\bar{x}$ ± s)	SARS‐CoV‐2 case			Normal reference range/unit
		Mild ($\bar{x}$ ± s)	Moderate ($\bar{x}$ ± s)	Severe ($\bar{x}$ ± s)	
WBC	8.241 ± 3.87	8.15 ± 4.2	8.29 ± 3.83	8.25 ± 3.71	4.4–11.9 × 10^9^/L
NEU	3.7624 ± 3.05	3.57 ± 3.22	3.22 ± 2.76	4.37 ± 3.10^a^***/^b^***	1.2–7.0 × 109/L
LYM	3.4044 ± 2.50	3.2879 ± 2.4^b^**	3.97 ± 2.40	2.96 ± 2.60^b^***	1.8–6.3 × 109/L
MO	1.23 ± 1.72	1.59 ± 2.94	1.12 ± 1.05	1.15 ± 1.36	0.12–0.93 × 109/L
NLR	2.32 ± 3.68	1.93 ± 2.44	1.58 ± 3.86^a^*	3.23 ± 3.94^a^**/^b^***	1–2
LMR	4.44 ± 4.16	3.98 ± 3.13^b^**	5.26 ± 4.48	3.91 ± 4.19^b^***	1.5–3.0
PLR	138.37 ± 128.55	136.92 ± 130.68	118.43 ± 115.07	157.29 ± 136.25^a^*/^b^***	110–400
PLT	285.88 ± 111.27	278.11 ± 97.66	314.74 ± 119.41^a^*	264.53 ± 105.84^a^*/^b^***	188–472 × 10^9^/L
CRP	13.78 ± 20.7	9.19 ± 11.80^b^***^/^ ^c^*	19.31 ± 29.96	12.78 ± 15.73	< 10 mg/L
ESR	17.63 ± 19.74	16.95 ± 18.95^b^*	24.34 ± 24.06	11.75 ± 12.49^b^***	0–20 mm/h
IL6	24.72 ± 29.98	23.1467 ± 20.91	22.74 ± 22.53	28.03 ± 40.36	0–10 pg/mL
PCT	0.39 ± 1.14	0.34 ± 0.84	0.23 ± 0.98	0.56 ± 1.37^a^***/^b^***	< 0.046 ng/mL
CK	155.99 ± 105.08	148.01 ± 88.12	139.16 ± 81.98	175.79 ± 127.28^a^**/^b^***	40–200u/L
CK‐MB	36.58 ± 21.76	35.36 ± 20.03	40.07 ± 23.06	34.11 ± 21.12^b^***	< 25u/L
LDH	333.43 ± 101.05	323.8 ± 97.33	349.6 ± 104.69^a^*/^c^**	322.99 ± 97.69	120–300u/L
PT	11.82 ± 1.61	11.85 ± 1.47	11.61 ± 1.32	12.02 ± 1.89^b^*	9.8–12.1 s
FIB	2.71 ± 1.05	2.55 ± 0.98^b^*	2.99 ± 1.21	2.51 ± 0.82^b^**	1.8–3.5 g/L
D‐D	18.76 ± 81.84	19.5 ± 72.07	1.42 ± 5.2	35.79 ± 116.17^b^**	< 0.55 mg/L
IgG	6.76 ± 2.75	6.06 ± 2.58	6.83 ± 2.78	7.04 ± 2.77	5.4–13.4 g/L

*Note:* Comparisons were made among the mild, moderate, and severe groups using one‐way analysis of variance (ANOVA) or the Kruskal–Wallis test.

Abbreviations: CK, creatine kinase; CK‐MB, creatine kinase isoenzyme; CRP, C‐reactive protein; DD, D‐dimer; ESR, erythrocyte sedimentation rate; FIB, fibrinogen; IgG, immunoglobulin G; IL‐6, interleukin‐6; LDH, lactate dehydrogenase; LMR, LYM‐to‐monocyte ratio; LYM, lymphocyte; MO, monocyte; NEU, neutrophil; NLR, neutrophil‐to‐lymphocyte ratio; PCT, procalcitonin; PLR, platelet‐to‐lymphocyte ratio; PLT, platelet; PT, prothrombin time; and WBC, white blood cell.

Data are expressed as the mean ± SEM (*p < 0.05; **p < 0.01; and ***p < 0.001).

^a^ Results compared with mild cases.

^b^ Results compared with moderate cases.

^c^ Results compared with severe cases.

#### 3.1.4 Correlation Analysis Between Clinical Indicators

In children with SARS‐CoV‐2 infection, WBC was positively correlated with PCT (*p* = 0.015); NEU was positively correlated with NLR (*p =* 0.005); NLR was negatively correlated with LDH (*p* = 0.019); PLT was negatively correlated with APTT (*p* = 0.042); PLR was negatively correlated with CK (*p* = 0.042); and IL‐6 was positively correlated with LDH (*p* = 0.019), PT (*p* = 0.005), and NLR (*p* = 0.042). The details can be seen in Table 4. The remaining results did not indicate any significant correlations and hence were not listed.

**TABLE 4:** Correlational analysis between clinical indicators.

Correlation between		Correlation coefficient	*p*
WBC	PCT	0.889	0.015
NEU	NLR	0.943	0.005
NLR	IL‐6	0.829	0.042
NLR	LDH	−0.886	0.019
PLT	APTT	−0.829	0.042
PLR	CK	−0.829	0.042
IL‐6	LDH	0.886	0.019
IL‐6	PT	0.943	0.005

*Note:* Spearman's correlation coefficient was applied to assess the correlations among WBC, NEU, NLR, PLT, PLR, and IL‐6 as variables and the liver, heart, kidney, coagulation function, and inflammatory indexes (PCT, ESR, and CRP), respectively.

Abbreviations: APTT, activated partial thromboplastin time; CK, creatine kinase; IL‐6, interleukin‐6; LDH, lactate dehydrogenase; NEU, neutrophil; NLR, neutrophil‐to‐lymphocyte ratio; PCT, procalcitonin; PLR, platelet‐to‐lymphocyte ratio; PLT, platelet; PT, prothrombin time; and WBC, white blood cell.

The significance value was set as *p* < 0.05.

### 3.2 Chest CT Analysis of SARS‐CoV‐2 Cases in Zhuhai City

Table 5 summarizes the chest CT imaging features of pediatric patients. Among the 124 pediatric patients who underwent the chest CT examination, 9 (1.2%) had normal imaging (i.e., no abnormality in both lungs) whereas 115 (15.6%) exhibited changes in chest CT imaging, suggesting statistically significant differences among all groups (*p* = 0.002). Abnormal chest CT findings included exudation and light spot shadows in 85 cases; pulmonary nodules, local emphysema, strip shadows, and mesh changes in 16 cases; vasculitis in 21 cases, ground glass opacity, uneven inflation, and mosaic changes in 14 cases; pleurisy and pleural effusion in 8 cases; lung consolidation and pneumobronchial signs in 9 cases; and bilateral lung texture changes without white lung‐like changes in 6 cases.

**TABLE 5:** Chest CT analysis of SARS‐CoV‐2 cases in Zhuhai City.

Characteristics	Total case (*n*)	SARS‐CoV‐2 case			*χ2/p*
		Mild (*n*)	Moderate (*n*)	Severe (*n*)	
Chest CT	124	5	52	67	
Normal (no abnormality in both lungs)	9	3	2	4	0.002
Abnormal	115	2	50	63	
Exudation and light spot shadows	85	0	39	46	
Pulmonary nodules, local emphysema, strip shadows, and mesh changes	16	0	6	10	
Vasculitis	21	0	7	14	
Ground glass opacity, uneven inflation, and mosaic changes	14	0	6	8	
Pleurisy or pleural effusion	8	0	4	4	
Lung consolidation and pneumobronchial signs	9	0	6	3	
Double lung texture change without white lung‐like changes	6	2	1	3	
White lung	0	0	0	0	

*Note:* The counting data were expressed as the number of cases and percentage, and the comparisons among the mild, moderate, and severe groups were made by the *χ2* test.

The significance value was set at *p* < 0.05.

## 4 Treatment and Regression

The treatment and clinical outcomes of SARS‐CoV‐2 patients are summarized in Table 6. A total of 157 (21.2%) patients received interferon antiviral therapy, whereas 309 (41.8%) patients were treated with antibiotics (Table 6). Among these, 268 (36.3%) patients received a single antibiotic and 41 (5.5%) patients received a combination therapy. The antibiotics used targeted common and some atypical pathogens, with adjustments made based on bacterial culture and drug sensitivity resulting in cases of secondary bacterial infections. Cephalosporins, carbapenems, macrolides (such as erythromycin and azithromycin), and linezolid for methicillin‐resistant *Staphylococcus aureus*, vancomycin, and antifungals were commonly administered.

**TABLE 6:** Treatment and prognosis of 739 cases of SARS‐CoV‐2 in Zhuhai.

Treatment and prognosis	Total case (*n*/[%])	SARS‐CoV‐2 case		
		Mild (*n*/[%])	Moderate (*n*/[%])	Severe (*n*/[%])
Case	739 (100%)	179 (24.2)	267 (36.1)	293 (39.7)
Treatment measure				
Antiviral (interferon)	157 (21.2)	43 (5.8)	64 (8.6)	50 (6.8)
Antibiotic	309 (41.8)	54 (7.3)	149 (21.2)	106 (14.3)
Alone	268 (36.3)	52 (7.0)	124 (16.8)	92 (12.5)
Combination	41 (5.5)	2 (0.3)	25 (3.3)	14 (1.9)
Chinese patent medicine	127 (17.2)	38 (5.1)	27 (3.7)	62 (8.4)
IVIG	17 (2.3)	2 (0.3)	1 (0.1)	14 (1.9)
Glucocorticoid	75 (10.2)	10 (1.4)	15 (2.0)	50 (6.8)
FiO2				
30% > FiO_2_ ≥ 21%	69 (9.3)	0	0	69 (9.3)
FiO_2_ ≥ 30%	19 (2.6)	0	0	19 (2.6)
Mechanical ventilation assistance				
Noninvasive	7 (0.9)	0	0	7 (0.9)
Invasive	4 (0.5)	0	0	4 (0.5)
Prognosis				
Cure	192 (26.0)	42 (5.7)	80 (10.8)	70 (9.5)
Remission	547 (74.0)	135 (18.3)	187 (25.3)	225 (30.4)
Death	0	0	0	0

*Note:* The counting data are expressed as the number of cases and percentage.

Abbreviations: FiO_2,_, fraction of inspiration oxygen and IVIG, intravenous immunoglobulin.

In addition, 127 (17.2%) patients received traditional Chinese medicines, including QingKailing Keli, Jinzhen Oral Liquid, Xiao'er Resuqing Granules, Xingbei Zhike Granules, Polygonati Odorati Rhizoma, Shedan Chuanbei Ye, Xiao'er Chaigui Tuire Keli, and Feilike Heji. Prednisone orally or intravenous methylprednisolone succinate or dexamethasone was administered to 75 (10.2%) patients, whereas 17 (2.3%) patients received intravenous immunoglobulin. Oxygen support was provided to 88 (11.9%) patients, with 69 (9.3%) patients receiving oxygen concentrations of fraction of inspired oxygen (FiO_2_, which is ≥ 21% and < 30%) and 19 (2.6%) patients receiving FiO_2_ ≥ 30%. Mechanical ventilation was required for 7 (0.9%) patients on noninvasive ventilation and 4 (0.5%) on invasive ventilator assistance; however, no patients underwent extracorporeal membrane oxygenation. Regression analysis showed that, after receiving anti‐inflammatory, symptomatic, supportive, and antipathogenic treatments, 547 (74.0%) patients achieved clinical remission and 192 (26.0%) patients were discharged as cured. Importantly, there were no fatalities among the patients.

## 5 Discussion

COVID‐19, categorized as a B‐class (i.e., generally a type of disease that has a high risk of transmission and is prone to widespread infection) infectious disease, has posed significant challenges since it emerged as a new type of infectious disease in late 2019 [5]. Children are particularly susceptible to respiratory droplet and contact transmission routes. Diagnosis primarily relies on epidemiological characteristics, clinical manifestations, pathogenicity, serological examinations, and chest imaging [3]. Currently, specific antiviral treatments are lacking, with symptomatic and supportive care being the mainstay to prevent complications.

The COVID‐19 pandemic has persisted for over 3 years, witnessing rapid mutation rates, leading to the emergence of variants, such as Omicron. Despite increased infectivity, subsequent strains have demonstrated diminished virulence and reduced lethality [5]. On December 7, 2022, the release of updated epidemic prevention and control measures marked a shift from “infection prevention” to “health protection and prevention of severe illness” [6]. However, the rapid relaxation of control measures at the onset of this new phase led to a resurgence in outbreaks, placing significant strain on the public health systems. This retrospective analysis focused on children infected during a large‐scale local epidemic in Zhuhai City, with the aim to provide insights into the prevention, identification of severe illnesses, and treatment strategies for SARS‐CoV‐2 infections in children. According to the current study, the pandemic affects children across all age groups. Our findings indicated that a majority of children admitted to hospitals with SARS‐CoV‐2 exhibited severe illness, with only a minority presenting mild symptoms with high‐risk factors (such as young age and basic disease) necessitating hospitalization. Consequently, our sample predominantly consisted of severely ill children (39.6%) rather than mildly ill children (24.2%), thereby diverging from earlier studies in which only mild cases predominated.

Consistent with previous research, our study underscores the association between the severity of SARS‐CoV‐2 infection in children and age distribution and underlying health conditions rather than sex distribution. Notably, children aged < 3 years accounted for a significant proportion (81.5%) of infections, particularly those < 1 year (43.2%). Currently, the inactivated novel coronavirus vaccine has been approved for the using in children aged ≥ 3 years. Our study found that SARS‐CoV‐2 infections in children aged > 3 years are less likely to progress to severe disease and that children aged ≤ 3 years are more susceptible to severe disease, particularly those aged 1–3 years. In our study, only 5.6% of the tested children had been vaccinated, with no statistically significant differences in the vaccination history and antibody IgG levels among all groups (*p* > 0.05). The low vaccination rate may be attributed to the majority of patients within 3 years of age, who have poor access to vaccines. This finding underscores that vaccination with the novel coronavirus vaccine continues to be an effective protective measure against infection with the Omicron variant strain, which aligns with the findings from several other studies [7, 8, 9]. Therefore, vaccination remains crucial for children aged > 3 years to mitigate the spread of Omicron variants. Whereas, with the increasing rates of infection and hospitalizations in children aged < 3 years, possibly due to the inadequate coverage of the novel coronavirus vaccine, vaccination should be made available even for children of age under 3 years to concur protection from severe COVID‐19 diseases [10].

Similarly, a clear history of exposure (63.1%) and family cluster morbidity (46.0%) were strongly linked to the onset and progression of SARS‐CoV‐2 infection in children. Children are notably susceptible to contracting the virus from family members, especially those under the age of 3 years, which highlights the need for robust family‐based prevention strategies and pediatric healthcare interventions. Furthermore, in the current phase of the epidemic, outbreaks linked to community exposure and clusters in educational settings are significant features of SARS‐CoV‐2 transmission among preschool and school‐aged children [7]. This finding highlights the imperative for stringent infection control measures across schools and childcare facilities for mitigating transmission risks among children.

Consistent with other studies, our research identified fever (91.7%) and cough (81.6%) as the most common clinical manifestations in children with SARS‐CoV‐2. Compared with earlier studies, the increased prevalence of nasal congestion (49.8%) and runny nose (53.7%) was noted in the present study, although clinical symptoms in children differed notably from those in adults [11]. Interestingly, moderate patients in our study exhibited prolonged fever, nasal congestion, runny nose, severe cough, and lung rales more frequently. Conversely, shortness of breath, convulsions, hypoxemia, and respiratory failure were significant indicators in severely ill children, providing crucial signals for timely disease management [3]. Drawing from previous research [12], the presence of gastrointestinal symptoms in SARS‐CoV‐2 patients may indicate more severe disease. However, our study found that, although a few children experienced symptoms, such as vomiting (10.6%), diarrhea (5.1%), and abdominal pain (3.2%), there was no statistical difference among the groups. Thus, gastrointestinal symptoms may not reliably differentiate severe cases during this phase of infection [13].

WBC count, LYM count, PLT count, and serum IL‐6 and ferritin levels serve as potential markers for predicting progression to critical illness [14, 15]. Furthermore, elevated serum IL‐6 and ferritin levels strongly correlate with disease severity [6, 16]. Biomarkers, such as PCT, NLR, LMR, and PLR, are also valuable in assessing infection and predicting disease severity [17]. Zhang et al. suggested that NLR outperforms NEU count alone in predicting the severity of COVID‐19 [18]. Henry and Huang et al. found that nonsurviving patients exhibited significantly higher NEU counts, serum CK, ferritin, and IL‐6 levels, along with significantly lower LYM and PLT counts, indicating an increased risk of clinical deterioration and poorer prognosis [16, 19]. Al Nafea and Wang et al. observed a significant correlation between increased coagulation indices and the severity of SARS‐CoV‐2 infection [20, 21]. Consistent with previous findings, our study recorded elevated NEU count, NLR, PLR, PT, and PCT, CK, DD, and IL‐6 levels in severe patients when compared with mild and moderate patients. Conversely, LYM count and PLT were lower in severe cases. In addition, IL‐6 was positively correlated with LDH, PT, and NLR. Interestingly, NLR was positively correlated with NEU, but negatively with LDH. It was thus speculated that IL‐6, as an important cytokine member, is highly expressed and involved in the abnormal inflammatory response associated with severe SARS‐CoV‐2 infection in children. In summary, these indicators appear to be closely linked to disease progression and poor prognosis in children with SARS‐CoV‐2 infection, with the potential to act as early biomarkers for predicting the progression of critical illness [22].

Chest CT examination is a convenient and rapid method for assessing the dynamic changes of COVID‐19 [23, 24]. The CT manifestations of COVID‐19 in pediatric patients vary widely and lack specificity, often correlating with the severity or clinical grade of the disease. In our study, a significant majority of patients (92.7%) exhibited notable lung CT abnormalities, with statistically significant differences (*p* = 0.002) observed among all groups. Particularly in severe cases, various degrees of lung lesions were evident on admission, including patchy infiltrates (37.1%) and prominent thickening of vascular bundles. Among the abnormal chest CT findings, the most common manifestation in children was exudative light spot shadows (68.5%), which represent the predominant lung abnormality in pediatric COVID‐19 cases. This pattern differs from that observed in adults [25, 26], potentially reflecting the generally milder presentation of SARS‐CoV‐2 in children compared to that in adults, and chest CT is not recommended as an early screening tool [25, 26, 27], but as an important test for the progression of severity.

The current therapeutic principles include anti‐inflammatory therapy, symptomatic therapy, supportive therapy, and antipathogen therapy [3]^.^ Interferon has both antiviral and immune‐regulating effects, with interferon‐α primarily used in clinical treatment for children with SARS‐CoV‐2 [3]. Recent research has indicated that early short‐term use of methylprednisolone improves clinical outcomes in moderate to severe cases of SARS‐CoV‐2 [28]. In addition, early oxygen support and low‐dose glucocorticoid therapy may help reduce mortality among severely ill patients. Traditional Chinese medicine has shown immunosuppressive properties that can be beneficial in preventing and treating cytokine storms [16], which play a critical role in managing SARS‐CoV‐2.

In the present study, we employed interferon antiviral therapy (21.2%), antibiotics (41.8%), traditional Chinese medicine (17.2%), low‐dose glucocorticoids (10.2%), immunoglobulin injection (2.3%), oxygen support therapy (11.9%), and mechanical ventilation (1.5%) for treatment. Following active treatment, 547 (74.0%) patients experienced symptom relief, 192 patients (26.0%) were discharged as cured, and there were no fatalities. This finding implies that children have a good prognosis with treatment, including antiviral, anti‐proinflammatory cytokines, anti‐infective, and immune‐supportive and life‐supportive therapies, especially in critically ill patients, and further confirms the reduced toxicity of the Omicron variant [5].

## 6 Limitations

This study has several limitations. First, the study was conducted over a short period of 1 month and 10 days. Second, the lack of data describing coinfections and secondary infection. Third, we were unable to assess any possible correlation between viral load and clinical symptoms. Fourth, because of the universal screening procedure used for inpatients, there was a selection bias in the collection of patients who were COVID‐19 positive but without respiratory symptoms. Thus considering that, they were not representative of the wider population of children.

## 7 Conclusion

In conclusion, our study revealed that most children infected with the Omicron variant of the novel coronavirus in Zhuhai were clustered due to close family contact, with nonspecific clinical manifestations. Young age, especially age < 1 year, the presence of underlying medical conditions, and lack of vaccination were identified as risk factors for developing severe infection. Several clinical indicators (including increased NEU count, NLR, PLR, PT, PCT, CK, DD, and IL‐6 levels and decreased LYM count, LMR, and PLT) were associated with the severity of SARS‐CoV‐2 infection. Early detection and monitoring of these indicators can potentially reduce disease progression, whereas addressing these factors may halt disease advancement. The most common chest CT finding was the exudation of light spots. Following treatment, the overall prognosis was favorable, further underscoring the reduced virulence of the Omicron variant.

## Author Contributions

**Ting Liu:** formal analysis, methodology, investigation and proofreading of first draft. **Zhaojun Pan:** formal analysis, investigation, data curation, writing – original draft, and visualization. **Cheng Zhang:** investigation. **Fanlin Huang:** investigation. **Jundong Ding:** investigation. **Wenxiu Song:** investigation. **Yongwu Xie:** conceptualization, methodology, resources, writing – review and editing, supervision, project administration, and funding acquisition. **Guang Lin:** conceptualization, methodology, resources, writing – review and editing, supervision, project administration, and funding acquisition.

## Ethics Statement

The study received an ethical review waiver from the Ethics Committee of Zhuhai Center for Maternal and Child Health Care, NO. 2024(102).

## Conflicts of Interest

The authors declare no conflicts of interest.

## Supporting information

Table S1

## Data Availability

The data that support the findings of this study are available from the corresponding author upon reasonable request.
