# Supplementary material for: Risk Factors and Clinical Features of Deterioration in 739 COVID‐19 Affected Children Aged Under 14 Years in Zhuhai, China: A Multicenter, Retrospective Study
Source: Pediatr Discov. 2025 May 19;3(2):e70001. doi: 10.1002/pdi3.70001 (PMC12258080; doi:10.1002/pdi3.70001)
Supplement: Supplementary file 1 — Table S1 [file PDI3-3-e70001-s001.docx]

**Table S1.** **Clinical Characteristics and** **Laboratory Results of 739 SARS-Co V-2 Cases in Zhuhai**

| Indicators | Total Case | SARS-Co V-2 case ( by disease severity and pathogen) | | | | *P* | Normal reference range/unit |
| --- | --- | --- | --- | --- | --- | --- | --- |
|  |  | Mild | Moderate | | Severe |  |  |
| Fever (d) | 2.77±1.97 | 2.40±1.60 | 3.41±2.32 | 2.39±1.63 | | *** | - |
| Cough | 603(81.6) | 131(17.7) | 254(34.4) | 218(15.2) | | *** | - |
| Nasal congestion | 368(49.8) | 82(11.1) | 163(22.1) | 123(16.6) | | *** | - |
| Runny nose | 397(53.7) | 89(12.0) | 165(22.3) | 143(19.4) | | ** | - |
| Sore throat | 128(17.3) | 44(6.0) | 13(1.7) | 71(9.6) | | *** | - |
| Convulsion | 191(25.8) | 0 | 0 | 191(25.8) | | - | - |
| Hoarseness | 138(18.7) | 29(3.9) | 39(5.3) | 70(9.5) | | * | - |
| Shortness of breath | 78(10.6) | 0 | 0 | 78(10.6) | | - | - |
| Vomiting | 78(10.6) | 25(3.4) | 27(3.7) | 26(3.5) | | - | - |
| Hypoxemia | 28(3.8) | 0 | 0 | 28(3.8) | | - | - |
| NEU | 3.7624±3.05 | 3.57±3.22 | 3.22±2.76 | 4.37±3.10^a***/b***^ | | - | 1.2-7.0×10^9^/L |
| LYM | 3.4044±2.50 | 3.2879±2.4^b**^ | 3.97±2.40 | 2.96±2.60^b***^ | | - | 1.8-6.3×10^9^/L |
| PLT | 285.88±111.27 | 278.11±97.66 | 314.74±119.41^a*^ | 264.53±105.84^a*/b***^ | | - | 188-472×10^9^/L |
| CRP | 13.78±20.7 | 9.19±11.80^b***/c*^ | 19.31±29.96 | 12.78±15.73 | | - | ＜10 mg/L |
| IL-6 | 24.72±29.98 | 23.1467±20.91 | 22.74±22.53 | 28.03±40.36 | | - | 0-10 pg/mL |
| PCT | 0.39±1.14 | 0.34±0.84 | 0.23±0.98 | 0.56±1.37^a***/b***^ | | - | ＜0.046 ng/ml |
| LDH | 333.43±101.05 | 323.8±97.33 | 349.6±104.69^a*/c**^ | 322.99±97.69 | | - | 120-300u/L |
| CK | 155.99±105.08 | 148.01±88.12 | 139.16±81.98 | 175.79±127.28^a**/b***^ | | - | 40-200u/L |
| D-dimer | 18.76±81.84 | 19.5±72.07 | 1.42±5.2 | 35.79±116.17^b**^ | | - | ＜0.55mg/L |

Note: The counting data were expressed as the numbers of cases and percentage, and *χ2* test was used for comparison of clinical characteristics between mild, moderate and severe groups, and one-way analysis of variance (ANOVA) or Kruskal Wallis test was used for comparison of laboratory results between mild, moderate and severe groups. a: results compared with mild cases; b: results compared with moderate cases; c: results compared with severe cases. Data are expressed as the mean ± SEM. *: *P* < 0.05; **: *P* < 0.01; ***: *P* < 0.001. neutrophil (NEU) , lymphocyte (LYM), platelet (PLT) ,C-reactive protein (CRP), interleukin-6 (IL-6), procalcitonin (PCT), lactate dehydrogenase (LDH), Creatine kinase (CK), and D-dimer (DD).
